# Osteo-Promoter Database (OPD) – Promoter analysis in skeletal cells

**DOI:** 10.1186/1471-2164-6-46

**Published:** 2005-03-25

**Authors:** Inbal Grienberg, Dafna Benayahu

**Affiliations:** 1 Department of Cell and Developmental Biology, Sackler School of Medicine, Tel-Aviv University, Israel

## Abstract

**Background:**

Increasing our knowledge about the complex expression of genes in skeletal tissue will provide a better understanding of the physiology of skeletal cells. The study summarizes transcriptional regulation factors interacting and cooperating at promoter regions that regulate gene expression. Specifically, we analyzed A/T rich elements along the promoter sequences.

**Description:**

The Osteo-Promoter Database (OPD) is a collection of genes and promoters expressed in skeletal cells. We have compiled a new viewer, OPD, as unique database developed and created as an accessible tool for skeletal promoter sequences. OPD can navigate to identify genes specific to skeletal cDNA databases and promoter analysis sites. OPD offers exclusive access to facilitate a dynamic extraction of promoters' gene-specific analyses in skeletal tissue. The data on promoters included in OPD contains cloned promoters or predicted promoters that were analyzed by bioinformatics tools. OPD offers MAR-analysis, which allocates A/T rich elements along these promoter sequences.

**Conclusion:**

The analysis leads to a better insight of proteins that bind to DNA, regulate DNA, and function in chromatin remodeling. The OPD is a distinctive tool for understanding the complex function of chromatin remodeling and transcriptional regulation of specific gene expression in skeletal tissue.

## Background

The Human Genome Project has provided large-scale sequencing and a multitude of genetic databases, based on information from bacteria, plant, *Drosophila*, vertebrates and mammals [1-5]. These databases represent evolutionary information that is summarized in the public databases NCBI, EMBL, and HUGO. *In silico *biology explores various tools that enable the translation of raw data into workable models and provides guidance for high-throughout gene analysis to 'make sense' of the genomic data. Computational tools analyzed the genetic information and data mining aided the knowledge interpretation in the functional cellular context. Genes expression from tissue-specific libraries can be developed to new models and add insight into biological information in a context of gene networks, protein pathways ect. Genes in eukaryotic cells are regulated in "active regions," in which chromatin structure is "open" and accessible to DNA-binding proteins and "silent regions" where "packed" chromatin renders the DNA inaccessible. The regulation is performed on several epigenetic levels that include DNA methylation, nucleosome positioning, histone modification, and additional components of higher-order structure. These modifications can activate or repress RNA synthesis that is associated with tissue-specific genes in the absence of transcriptional enhancers. The details of how chromatin structures modified are unresolved, but it is clear that DNA methylation has a direct effect on both histone acetylation and on higher-order chromatin structure that regulates DNA-chromatin structure [6].

Transcriptional regulation factors interacting and cooperating at promoter regions determine the fate of cells and their function. The promoter is a cis-acting element immediately upstream of the transcription start site (TSS) that controls the rate of the initiation of transcription. The promoter region is important in regulation of various processes in development, morphogenesis, cell differentiation; hormonal communication and cellular stress responses. To date, only a small fraction of promoters is identified and most are not yet cloned. To better understand tissue specificity, there is a need to explore the interactions that occur between chromatin remodeling proteins and the promoters. Such interactions take place next to transcription-factor (TF) binding sites. An important motif presented in the promoter is an A/T-rich region that binds proteins with an A/T-hook motif. Numerous chromatin-remodeling proteins identified by presence of single or multiple A/T hook motifs that form complex of the protein with the DNA [7]. The A/T rich regions are characteristic by MAR frequently observed at the genomic regions and affect the interactions with the A/T hook motif of the proteins [8].

The knowledge evolve transcription factor binding sites on the promoter will allow the creation of the regulation map of each gene and will enable the recognition of other transcription regulation systems that control gene expression. To learn how promoters operate, it is first necessary to identify them. To date, a relatively small number of promoters have been identified, and, thus, it is possible to predict the location of the promoter upstream to the coding regions of the gene sequence. Gene-structure prediction programs, such as BCM Gene Finder tool, ConPro [9], Core Promoter [10], FirstEF [11], Gene2Promoter [12], FunSiteP [13] and MatInspector [14] enable to classify promoters. Promoters are being analyzed on species levels as well, for example, the *Saccharomyces *promoter database (SCPD) [14,15] and *C. elegans *promoter database (CEPDB) , EPD is a Eukaryotic Promoter Database [16], and LSPD is a liver-specific tissue promoter database . Transcription factors bind to regulatory elements upstream of transcription start sites and interact with other factors to create an abundant regulatory environment. Each regulatory protein binds to a specific element on the promoter, in response to an appropriate signal. The complexity of the regulatory system can be explored by the use of software that searches for specific transcription elements on the promoter. Genomatix offers S/MARt DB [17] and S/MARs [18], aid in the analysis of scaffold/matrix-attached regions and the nuclear matrix proteins.

### Construction and content

The OPD homepage contains a list of genes, where each gene has one entry link that enables the user to obtain the specific gene description. Skeletal genes which are cataloged in OPD are obtained from Human Bone libraries such as: Skeletal Gene Database (SGD) [19] and Human Bone Marrow stroma (lib. 931) [20]. Bone tissue specific expression was defined using the Unigene database . Information about gene products, their cellular function and chromosomal locus imported from the NCBI database. Accession numbers were used to link the gene entries to information in the resource databases. Accession numbers were obtained from NCBI and HUGO. Information about human genetic disorders and their associated genes is derived from the Online Mendelian Inheritance in Man database, OMIM . All the references were structured with hyperlinked both to the original Web servers and to the NCBI and HUGO database and OMIM. Searching for a gene's promoter was performed using NCBI and PUBMED databases. The accession number of each promoter is linked to the promoter sequence page in the NCBI database. MAR-Analysis in the promoter region is carried out using the MAR-FINDER program [21]. The MAR Finder program algorithm scores A/T nucleotides along promoters using a 200 bp window at 10 bp intervals with MAR potential of 0.1 as minimum significance threshold.

### Utility; Osteopromoter Database (OPD) description

The Osteo-Promoter Database (OPD) summarizes the information about genes and promoters were designed based on analysis of resources covering skeletal cells. The functional genes presented in OPD were annotated and catalogued from human bone marrow stroma [19] and the skeletal gene database (SGD) [20]. The OPD is a novel bone-tissue promoter database that includes hundreds of genes that mediate osteoblast cells proliferation and differentiation [19,20,22-25]. The genes and promoters included in OPD from the PUBMED, bibliographic references databases, and cDNA sequences centers and more promoters that were identified using Gene2Promoter. The data is organized in alphabetical order and have cross-references with other databases.

In OPD, we summarized series of genes known to play a role in the regulation of skeletal cells. The information explores the transcription controlled by various factors on skeletal cells [22-25]. OPD presents distribution of transcription-factor sites in promoter regions and the connection with chromatin structure [26,27]. Promoters were analyzed and enlighten on the connections between transcription factors and chromatin remodeling factors. We choose to focus on A/T-rich region that binds A-T hook proteins and serves as interface between transcription factors and chromatin remodeling proteins. MAR regions that contain A/T-rich elements are embedded along the genome and recognized for their role in the regulation of gene transcription by creating an environment that controls gene expression at the chromatin remodeling level.

Specifically, examples are given to genes related to progenitors proliferate and differentiate to osteoblasts that form the calcified matrix. Table 1 summarizes genes related to cell proliferation, differentiation and matrix deposition in the formed bone. Transcription factors and hormones control the expression of genes responsible for cell proliferation. The cell's differentiation requires sequential activation of regulatory proteins and signaling molecules such as BMPs and hormones. The osteoblast maturation is defined by the biosynthesis and organization of extracellular matrix proteins, such as osteocalcin, bone sialoprotein and osteonectin [23]. We identified from the literature two hundred promoters' that were cloned, and the rest of the promoters included in OPD, were not cloned but identified using bioinformatics tools.

**Table 1:** Genes related to cells stages of differentiation

***Proliferation***	***Differentiation***	***Mineralization***
Dlx5	Collagen 2A1, Collagen 1A1	phosphatase Alkaline
jun-c, fos-c	BMP7, BMP4, BMP2	Osteopontin
Cbfa1	TGFR beta3, TGFR beta1	Osteocalcin
Vitamin D	IGF2, IGF1	sialoprotein Bone
Prostaglandin E2	Fibrillin 1, 2	Osteonectin
PTH	FGER 1-3	Biglycan

Table summarized gene expressed by cells at different stages of differentiation. Genes are related to various families of functional genes; growth factors, transcription factors and hormones are presented according their expression throughout osteoblastic differentiation.

### Database organization

#### Home Page

**Figure 1:**
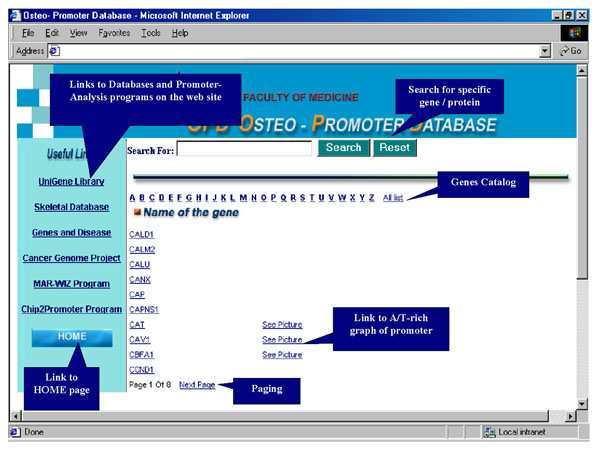
OPD catalogs hundreds of genes and promoters that are organized in alphabetical order, with direct access from the OPD homepage to each specific gene possible via the list. A search for a specific protein can be carried out by paging the list or by using the search engine on the top of the Homepage. Each name of gene is linked to gene presentation. Clicking on "see picture" link enables entry to MAR analysis of each promoter. From the page are links to the promoter analysis and to the other genomic databases sites.

**Figure 2:**
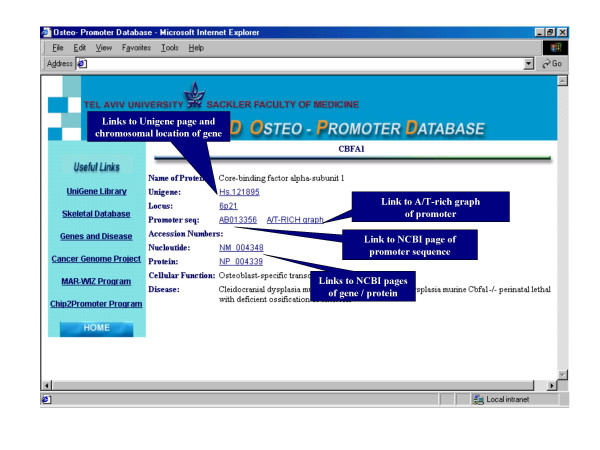
Entry into the gene page will provide a description for specific gene, including expression analysis and accession numbers structured as specific links. The description includes also cellular function and linked disease for mutations when available. An example of the Cbfa1 gene is presented and the promoter sequence linked to the NCBI page is shown. MAR analysis of this promoter is performed through "A/T RICH graph" link. Accession numbers are linked to NCBI and UNIGENE pages, which present gene /protein sequences and bibliographic references.

OPD catalogs hundreds of genes and promoters that are organized in alphabetical order, with direct access from the OPD homepage to each specific gene possible via the list. To view a specific gene, one can view the list and choose the gene or alternatively, identify the gene through a search engine. To search for the gene, the user types the full name of the gene/protein into the search engine box and clicks on the "search" button (Fig. 1). Entry into the gene page will provide a description for each gene, including expression analysis and accession numbers structured as specific links. The information for each gene/protein on the promoter includes chromosomal location, catalog international number of sequences, cellular function, mutations (OMIM), and involvement in differentiation or diseases (Fig. 2, Table 2). A/T-rich region analyses are available through the "see picture" link next to the name of the gene. Absence of a link means that promoter of the gene has not been investigated yet. On the left side of the page, there are useful links to the promoter analysis and to the genomic databases sites (Fig. 1). The OPD presents promoter sequences identified and readable from positions within sequences of the HUGO and NCBI databases. Other links to skeletal cDNA databases and promoter analysis sites are presented.

**Table 2:** Detailed information related to genes in osteogenic differentiation

GENE	PROTEIN	CELLULAR FUNCTION	DISEASE	LOCUS	ACCESSION NUMBER	PROMOTER
BGLAP	Bone gamma-carboxyglutamate (gla) protein (osteocalcin)	Gamma-carboxyglutamic acid residues are formed by vitamin K dependent carboxylation. These residues are essential for the binding of calcium.		1q25-q31	**P**-NP_000702 **N**-NM_000711 **U**-Hs.2558	AY147065
BGN	Biglycan	Binding to collagen fibrils and transfering growth factor-beta. It may promote neuronal survival.	Happle syndrome	Xq28	**P**-NP_001702 **N**-NM_001711 **U**-Hs.821	U82940 X83526
BMP4	Bone morphogenetic protein 4	Involved in bone induction & tooth development	Fibrodysplasia ossificans progressiva	14q22-q23	**P**-NP_001193 **N**-NM_001202 **U**-Hs.68879	AH007194 AF210053
CBFA1	Core-binding factor alpha-subunit 1	Osteoblast-specific transcription factor	Cleidocranial dysplasia murine Cbfa1+/- cleidocranial dysplasia murine Cbfa1-/- perinatal lethal with deficient ossification of skeleton	6p21	**P**-NP_004339 **N**-NM_004348 **U**-Hs.121895	AB013356
ESR1	Estrogen receptor 1 (ER alpha)	Activities of this protein have been demonstrated in the regulation of a variety of genes including lactoferrin, osteopontin, medium-chain acyl coenzyme A dehydrogenase (MCAD) and thyroid hormone receptor genes.		6q25.1	**P**-NP_004442 **N**-NM_004451 **U**-Hs.110849	X62462
PRLR	Prolactin receptor	This is a receptor for the anterior pituitary hormone prolactin		5p14-p13	**P-NP_000940** **N**-NM_000949 **U**-Hs.1906	AF091859

List of factors such as transcription factors, hormones and matrix proteins play a role mediating of osteoblast differentiation.

#### Management System

The database is constructed based on Access database and is presented on the Web pages were created dynamically by ASP scripts written in html and VB script language. The A/T-rich graphs designed in Bitmap format.

### Promoter analysis

OPD summarizes genes that are important in the control of osteoblast differentiation. OPD includes hundreds of promoters that were identified by cloning and the study of the promoter function was performed at the molecular level. Other promoters included were not cloned, but are related to genes relevant to skeletal-cell differentiation and function, which we investigated based on each promoter's prediction from the genomic region. The task of OPD focused on resolving A/T-rich sequences in a defined sequence, using the MAR-FINDER algorithm. The analysis by MAR-FINDER is based on the integration of the frequency of the motif in a defined sequence in relation to the probability of their random occurrence in the same sequence. This software scores A/T nucleotides in a given sequence, and statistical significance is associated with the frequency of occurrence of the pattern influenced by A-T content.

Using bioinformatics we predicted promoter structures from genomic contigs that had not been cloned and followed with the analyzed of A/T-rich elements. Herein we present few examples presented at the OPD; TGFβ3 and TGFRβ3 promoters were predicated. TGFβ3 is constructed from 7 exons and the promoter region upstream to TSS in contig NT_026437 identified A/T rich at -1180-1970 bp. TGFRβ3 gene was identified in contig NT_034388 includes 18 exons and the promoter was identified from the same genomic region, includes three A/T-rich elements at -420-1120 bp, -1250-1330 bp, and 1690-1970 bp. An analysis for BMP factors that are part of the TGF-β superfamily know for their role in regulating the growth and differentiation of osteoblasts. Promoters for BMP2, BMP4, and BMP7 were analyzed and identified the A/T-rich element for the BMP2 at -250-820 bp, and for BMP4 promoter three A/T-rich elements were identified at 250–430 bp, 670–930 bp, and 1170–2150 bp, but none were identified for BMP7. The BMP4 promoter includes three A/T-rich elements with high MARs potentials and thus suggests a significant potential to bind protein with an A-T hook motif.

The differences among MAR-potential are reflected by the percentage of A/T-rich sequences, higher MAR regions suggests a higher potential for binding of a protein with an A-T hook domain. MARs play a role in the context of long-distance interactions mediated between locus control regions (LCR) and the target promoters by creating loops of DNA. The DNA loop keeps the flexibility in A/T-rich length for its functionality. The presence of several copies of A/T-rich sites and longer stretches of A/T-rich sequence create a wider surface area for bonding a specific protein [28,29]. HMG-I/Y protein contains three A-T hooks responsible for multivalent interactions between the A-T hooks and appropriately spaced A/T-rich tracts are required for high-affinity binding. A single A-T hook in a polypeptide or a single A-T tract on the target DNA results in reduced interaction [28]. Furthermore, the C-terminal acidic region and the spacer regions between A-T hooks also affect the protein – DNA binding [29].

Fibroblast growth factors (FGFs) are regulators of cells division and differentiation [26] also control alkaline phosphatase; and regulate matrix proteins synthesis such as collagen, osteonectin, and osteocalcin. Four FGF receptors (FGFR 1–4) members of the tyrosine kinase receptors family were identified. FGFR1 and FGFR2 promoters were predicted and analyzed for MAR region results with two A/T-rich sites with high MAR potential for FGFR1 and lower for FGFR2. These different MAR potentials expressed in the promoters of functional genes assumed for the potential of protein to bind at a different affinity in these promoters. The analysis of promoters related to functional genes in osteogenic differentiation results with annotated genes extended for analysis by MAR-FINDER regions (Table 2). The results have shown that 73% of these genes expressing MARs regions suggesting that MAR element are a common regulatory element in transcriptional control. Thus, it indicates for A/T-rich regions a role in control and guidance of activation and repression of osteoblastic differentiation. The accessibility of proteins to A/T-rich sites on promoters of tissue-specific genes along with other factors will promote the comprehensive specificity of the regulatory process of functional genes in bone tissue.

The presence of several A/T-rich sites on promoters and their high MAR potential implies for their involvement in regulation by creating the boundaries for protein interactions. Along the human genome, 100,000 MAR regions contain A/T-rich elements. The MAR regions were recognized along with other regulatory elements at the promoters, such as enhancers and transcriptional-factors, binding sites that form protein-complex regulators for DNA transcription [18]. MAR regions control expression of genes, since they subdivide the chromatin into DNA loops, which mediate between Locus controls regions (LCRs) to distant promoters [30]. Frequency of motifs in MAR elements are influenced by the A/T content [8] that participates in global transcription regulation by binding RNA polymerase to the nuclear matrix and creating the regulatory structure necessary for transcriptional initiation. Thus, the interaction between A/T-rich sites on the DNA to A/T-hook motif of the protein is an important component of the regulatory complex that controls transcription of genes [7].

## Conclusion

The data presented in OPD will assist in the study of gene regulation by proteins that possess A/T-hook motifs. The OPD provides a broad analysis of the promoters essential for skeletal cell regulation and an accessible resource for the skeletal research community. This analysis will deepen and facilitate our understanding of the mechanism of transcriptional regulation of osteogenic cells. The construction of OPD, a bone-specific promoter database, will lead to the development of a novel skeletal-enhanced promoter-based microarray for studying gene expression in skeletal tissues at different stages of growth, development and the pathological state of malignancies and metabolic bone diseases.

## Availability and requirements

Osteo-Promoter Database (OPD), available through web at 

## Authors' contributions

GI carried out the collections of genes in OPD, made the predication of genes' promoters, constructed the web site and drafted the manuscript. BD participated in the design of the study, conceived of the study, coordination and helped to draft the manuscript. All authors read and approved the final manuscript.

## References

[B1] Rombauts S, Dehais P, Van Montagu M, Rouze P (1999). PlantCARE, a plant cis-acting regulatory element database. Nucleic Acids Res.

[B2] Higo K, Ugawa Y, Iwamoto M, Korenaga T (1999). Plant cis-acting regulatory DNA elements (PLACE) database: 1999. Nucleic Acids Res.

[B3] (1999). The FlyBase database of the Drosophila Genome Projects and community literature. The FlyBase Consortium. Nucleic Acids Res.

[B4] Blake JA, Eppig JT, Richardson JE, Davisson MT (2000). The Mouse Genome Database (MGD): expanding genetic and genomic resources for the laboratory mouse. The Mouse Genome Database Group. Nucleic Acids Res.

[B5] Strausberg RL, Feingold EA, Klausner RD, Collins FS (1999). The mammalian gene collection. Science.

[B6] Geiman TM, Robertson KD (2002). Chromatin remodeling, histone modifications, and DNA methylation-how does it all fit together?. J Cell Biochem.

[B7] Aravind L, Landsman D (1998). AT-hook motifs identified in a wide variety of DNA-binding proteins. Nucleic Acids Res.

[B8] Liebich I, Bode J, Reuter I, Wingender E (2002). Evaluation of sequence motifs found in scaffold/matrix-attached regions (S/MARs). Nucleic Acids Res.

[B9] Liu R, States DJ (2002). Consensus promoter identification in the human genome utilizing expressed gene markers and gene modeling. Genome Res.

[B10] Zhang MQ (1998). Identification of human gene core promoters in silico. Genome Res.

[B11] Davuluri RV, Grosse I, Zhang MQ (2001). Computational identification of promoters and first exons in the human genome. Nat Genet.

[B12] Scherf M, Klingenhoff A, Werner T (2000). Highly specific localization of promoter regions in large genomic sequences by PromoterInspector: a novel context analysis approach. J Mol Biol.

[B13] Hehl R, Wingender E (2001). Database-assisted promoter analysis. Trends Plant Sci.

[B14] Quandt K, Frech K, Karas H, Wingender E, Werner T (1995). MatInd and MatInspector: new fast and versatile tools for detection of consensus matches in nucleotide sequence data. Nucleic Acids Res.

[B15] Zhu J, Zhang MQ (1999). SCPD: a promoter database of the yeast Saccharomyces cerevisiae. Bioinformatics.

[B16] Praz V, Perier R, Bonnard C, Bucher P (2002). The Eukaryotic Promoter Database, EPD: new entry types and links to gene expression data. Nucleic Acids Res.

[B17] Liebich I, Bode J, Frisch M, Wingender E (2002). S/MARt DB: a database on scaffold/matrix attached regions. Nucleic Acids Res.

[B18] Frisch M, Frech K, Klingenhoff A, Cartharius K, Liebich I, Werner T (2002). In silico prediction of scaffold/matrix attachment regions in large genomic sequences. Genome Res.

[B19] Jia L, Young MF, Powell J, Yang L, Ho NC, Hotchkiss R, Robey PG, Francomano CA (2002). Gene expression profile of human bone marrow stromal cells: high-throughput expressed sequence tag sequencing analysis. Genomics.

[B20] Jia L, Ho NC, Park SS, Powell J, Francomano CA (2001). Comprehensive resource: Skeletal gene database. Am J Med Genet.

[B21] Singh GB, Kramer JA, Krawetz SA (1997). Mathematical model to predict regions of chromatin attachment to the nuclear matrix. Nucleic Acids Res.

[B22] Katagiri T, Takahashi N (2002). Regulatory mechanisms of osteoblast and osteoclast differentiation. Oral Dis.

[B23] Aubin JE (2001). Regulation of osteoblast formation and function. Rev Endocr Metab Disord.

[B24] McCarthy TL, Ji C, Centrella M (2000). Links among growth factors, hormones, and nuclear factors with essential roles in bone formation. Crit Rev Oral Biol Med.

[B25] Karsenty G (2001). Minireview: transcriptional control of osteoblast differentiation. Endocrinology.

[B26] Bucher P, Trifonov EN (1986). Compilation and analysis of eukaryotic POL II promoter sequences. Nucleic Acids Res.

[B27] Bucher P (1990). Weight matrix descriptions of four eukaryotic RNA polymerase II promoter elements derived from 502 unrelated promoter sequences. J Mol Biol.

[B28] Maher JF, Nathans D (1996). Multivalent DNA-binding properties of the HMG-1 proteins. Proc Natl Acad Sci U S A.

[B29] Frank O, Schwanbeck R, Wisniewski JR (1998). Protein footprinting reveals specific binding modes of a high mobility group protein I to DNAs of different conformation. J Biol Chem.

[B30] Heng HH, Krawetz SA, Lu W, Bremer S, Liu G, Ye CJ (2001). Re-defining the chromatin loop domain. Cytogenet Cell Genet.

